# Accuracy of Deep Learning Models in Detecting Mandibular Furcation Defects on Panoramic Radiographs

**DOI:** 10.3390/diagnostics16101500

**Published:** 2026-05-15

**Authors:** Meric Kurumlu, Fatma Karacaoglu, Mürüvvet Kalkan, Irem Ulku, Erdem Akagunduz, Kaan Orhan

**Affiliations:** 1 Department of Periofontology, Faculty of Dentistry, Ankara University, 06560 Ankara, Turkey; 2 Department of Computer Engineering, Ankara University, 06830 Ankara, Turkey; 3 Department of Modelling and Simulation, Graduate School of Informatics, Middle East Technical University, 06800 Ankara, Turkey; 4 Department of Dento Maxillofacial Radiology, Faculty of Dentistry, Ankara University, 06560 Ankara, Turkey; 5 Department of Oral Radiology, School and Hospital of Stomatology, Cheeloo College of Medicine, Shandong University, Jinan 250012, China; 6 Medical Design Application and Research Center (MEDITAM), Ankara University, 06230 Ankara, Turkey

**Keywords:** artificial intelligence, diagnostic imaging, periodontal disease, computer-assisted diagnosis, dental radiography, periodontitis

## Abstract

**Background/Objectives**: Furcation defects pose a significant challenge in the diagnosis and treatment planning of periodontal diseases. Accurate clinical identification of furcation involvement is essential for improving treatment outcomes. This study aimed to evaluate the accuracy and effectiveness of various artificial intelligence (AI) algorithms in detecting furcation defects (FD) in mandibular molars. **Methods**: A total of 654 panoramic radiographs were randomly selected from patients who visited the Department of Oral and Maxillofacial Radiology at the Faculty of Dentistry, Ankara University. Each image was labeled as either “healthy” or “FD” and subsequently preprocessed. The performance of different deep learning algorithms in identifying FD was subsequently evaluated. **Results**: In the classification models employed, the highest scores were calculated as accuracy 97.9%, precision 97.10%, sensitivity 97.08%, and F1 score 97.09% in the Xception model. In the segmentation tests, the highest scores were calculated as accuracy 99.96%, precision 99.26%, sensitivity 97.57%, and F1 score 98.41% in the ENet model. **Conclusions**: Results of this study indicated that the use of artificial intelligence systems in detecting furcation involvement in mandibular molar teeth in panoramic radiography images is promising. Further studies covering larger data sets, including maxillary molar teeth, will increase the success rates in detecting furcation involvement.

## 1. Introduction

Periodontitis is a chronic, multifactorial disease characterized by periodontal pocket formation, loss of periodontal supporting tissues, clinical attachment loss, and radiographic bone loss. If left untreated, periodontal inflammation may progress to severe attachment loss, particularly affecting the bifurcation or trifurcation areas of multirooted teeth [1]. Accurate identification and assessment of periodontal destruction in these regions are essential for effective treatment planning. Although radiographs alone are often insufficient for diagnosis of furcation involvement, their correlation with clinical findings plays a crucial role in achieving an accurate diagnosis [1]. Radiographic evaluation of the furcation area can be performed using intraoral techniques, panoramic radiography, and cone-beam computed tomography (CBCT). While panoramic radiography has certain limitations, such as lower resolution, reduced detail, and superimposition of anatomical structures compared to intraoral techniques, it offers time efficiency and reduced radiation exposure when compared to a full-mouth intraoral radiographic examination [2].

The concept of ‘artificial intelligence’ refers to the ability of machines to perform tasks that are typically carried out by humans. Different techniques are used in AI, like machine learning, deep learning algorithms, image classification, and segmentation. Deep learning algorithms are designed to process data, extract features, and learn complex relationships using artificial neural networks structured in multiple layers [3]. Convolutional neural networks (CNNs), which employ a specialized architecture for processing image data and extracting relevant features, have demonstrated remarkable success in image-based applications [4,5,6]. Owing to their architectural design, CNNs are capable of addressing complex visual challenges and delivering results with high accuracy. In the context of disease detection, these networks enable early diagnosis by efficiently analyzing medical images [7,8].

In recent years, advanced computer technologies, particularly artificial intelligence (AI), have started playing a role in various fields of dentistry, contributing to solutions for the growing need for diagnostic and treatment solutions [9,10,11]. High accuracy rates have been reported in studies employing AI for the diagnosis of periodontal diseases, particularly the assessment of periodontal and peri-implant bone loss, detection of periodontal defects, and disease staging [12,13,14,15,16,17].

The aim of this study is to systematically evaluate and compare the performance of multiple deep learning-based classification and segmentation models for detecting mandibular furcation defects on panoramic radiographs, and to assess their potential applicability as reliable clinical decision-support tools.

## 2. Materials and Methods

This study was conducted with a dataset consisting of panoramic images obtained from the Faculty of Dentistry, Ankara University. It was approved by the Non-Interventional Clinical Research Ethics Committee (3 July 2023, number 36290600/36/2023, Clinical Research Ethics Committee of Ankara University Faculty of Dentistry) and complies with the principles of the Declaration of Helsinki. In this study, panoramic radiographs of individuals aged over 18 years with adequate image quality were randomly selected for inclusion in the study. Radiographs with poor image quality, as well as those obtained from pediatric patients, were excluded from the analysis. A total of 654 panoramic radiographs from 654 patients were included in the study.

The panoramic radiographs were uploaded in TIFF format to Adobe Photoshop 2023 (version 24.0). Photoshop was specifically utilized because it allows for precise, manual, pixel-level delineation of anatomical borders. The furcation areas of multi-rooted teeth were carefully examined, and radiolucent areas indicative of bone resorption were identified and labeled as furcation defects. This labeling process was performed entirely manually by tracing the root boundaries of the affected teeth and delineating the corresponding lesions, without the use of any automated or machine-assisted methods. All images were independently evaluated by two periodontologists (FK and MK). Upon completion of the labeling process, the dataset was re-evaluated by the observers, and any discrepancies were resolved through consensus. In order to assess intraobserver reliability, 35 panoramic radiographic images were re-evaluated one month after the initial analysis. The intraobserver correlation coefficient was calculated as 0.80, indicating a strong level of agreement.

In the study, pre-trained convolutional neural network architectures were used to classify panoramic radiograph images as either patient or healthy to evaluate the effectiveness of deep learning in detecting furcation defects.

### 2.1. Preparation for Training the Classification Model

The following steps were followed to prepare the classification model for training:

Each panoramic radiograph was divided into eight cropped regional images. Crops that did not contain diagnostically meaningful tooth or furcation-related information were excluded from the dataset. After this filtering process, the classification dataset consisted of 4192 cropped images in total, including 2096 furcation-positive images and 2096 furcation-negative healthy images (Table 1).

**Dataset Splitting:** The dataset was partitioned into three subsets: 70% for training, 20% for validation, and 10% for testing purposes. The split was performed at the cropped-image level rather than at the patient level; therefore, the unit of randomization was the cropped image. For the classification task, the training set included 2934 cropped images, consisting of 1467 furcation-positive and 1467 furcation-negative images. The validation set included 838 cropped images, consisting of 419 furcation-positive and 419 furcation-negative images. The test set included 420 cropped images, consisting of 210 furcation-positive and 210 furcation-negative images.

**Preprocessing:** Each image was transformed into one-dimensional matrices representing RGB values and subsequently normalized to a range of [0, 1].

**Models:** Pre-trained models obtained from the TensorFlow library were used as base models. These models consisted of multiple layers [4].

**Prediction Layer:** The softmax activation function was applied to generate the output of the model, providing the most likely prediction for each class.

Model Training: Deep learning architectures including UNet, Inception UNet, SegNet, E-Net, Xception, ResNet152V2, MobileNetV2, and Inception V3 were employed. Following training, the models were evaluated using accuracy, precision, recall, and F1-score metrics, along with categorical cross-entropy loss. The primary model was trained for 20 epochs.

**Model Evaluation:** The performance was evaluated by examining the metrics obtained from predictions on the test dataset. The evaluation included accuracy, cross-entropy loss, precision, recall, and F1-score. These results were later used to compare the overall performance of the main models.

### 2.2. Preparation for Training the Segmentation Model

For the segmentation task, only the furcation-positive cropped images were used. The furcation region was annotated on each positive cropped image, resulting in 2096 RGB cropped images and 2096 corresponding binary mask images.

**Image Preparation:** Images were resized to a consistent dimension of 224 × 224 pixels.

**Data Augmentation and Splitting:** The dataset was divided into training, validation, and testing sets at a ratio of 70:20:10, respectively. For the segmentation task, the training, validation, and test sets included 1467, 419, and 210 image–mask pairs, respectively (Table 1).

**Model Preparation:** One of the UNet, SegNet, or Inception UNet architectures was selected and used for the segmentation process. Encoder–decoder connections were established accordingly.

**Training:** The selected segmentation models were trained for 20 epochs using a batch size of 32 and a learning rate of 1 × 10^−4^. The Adam optimizer was used during training. Additionally, 5-fold cross-validation was applied to ensure the robustness of the model. Loss values and performance metrics were recorded at each epoch to monitor training progress.

**Model Evaluation:** The trained models were evaluated using a dedicated test dataset not included in the training process. Final performance metrics were calculated to assess segmentation accuracy.

**Figure 1:**
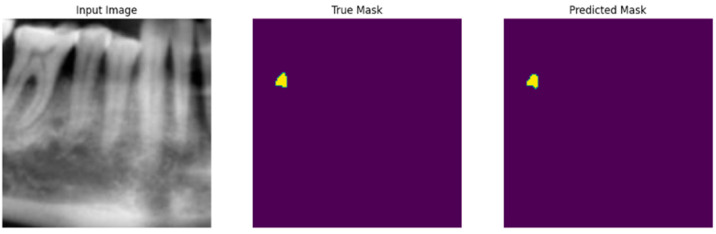
The sample prediction results are presented as follows: first, the original image along with its file name; second, the ground truth mask corresponding to the defect region; and finally, the predicted mask generated by the segmentation model.

**Visualization:** For qualitative assessment, the input image, corresponding ground truth mask, and the predicted mask were visualized for the test dataset, as illustrated in Figure 1.

In the experiment, the Google TPU V2 processing unit was utilized. Predictions were obtained in the form of 1D arrays or vectors through the use of the softmax function as the activation function in the final prediction layer. This allowed for categorical classification. After the classification process was completed and the test images were identified, an input image belonging to a patient was provided for segmentation purposes.

### 2.3. Evaluation Metrics

To assess the performance of all classification models, the following evaluation metrics were utilized: accuracy, precision, recall (sensitivity), F1-score, and categorical cross-entropy loss. For segmentation models, the Jaccard Index (Intersection over Union) was also calculated to provide a more detailed assessment of the predicted masks. In the context of binary classification, predictions were categorized as follows based on the ground truth:

TP (True Positives): Number of positive defects correctly predicted.

FP (False Positives): Number of negative cases incorrectly predicted as positive.

TN (True Negatives): Number of negative defects correctly predicted.

False Negatives (FN): The number of defects that are actually positive but incorrectly predicted as negative.

Precision

Quantitatively expresses the ratio of the model’s true positive predictions (TP) to the total predicted positives (TP and FP). Specifically, as presented in the equation, it evaluates how accurately the model detects actual positive defects.

Precision = TP/(TP + FP)

Sensitivity (Recall)

As presented in the equation, sensitivity (recall) represents the proportion of true positive samples (TP) correctly identified by the model over all actual positives (TP + FN).

Recall = TP/(TP + FN)

F1 Score

The F1 score is critical when there is a significant imbalance between precision and recall, and a balance between these metrics is needed. It is particularly useful in minimizing false predictions.

F1 = 2 × (Precision × Recall)/(Precision + Recall)

Alternatively,

F1 = TP/(TP + ½ (FP + FN))

Jaccard Index

The Jaccard Index measures the ratio of the intersection area to the union area of two sets during the segmentation phase. The Jaccard Index ranges from 0 to 1; a value of 1 indicates complete overlap between the sets, while 0 indicates no overlap. A high Jaccard Index demonstrates a significant agreement between the model’s predicted segments and the ground truth segments.

Jaccard Index = |A ∩ B|/|A ∪ B| = TP/(TP + FP + FN)

## 3. Results

The performance metrics of the classification models employed in our study are presented in Table 1. In the MobileNet V2 model, accuracy was 96.42%, precision was 96.59%, recall was 96.44%, and the F1 score was 96.52%. In the Xception model, accuracy was 97.9%, precision was 97.10%, recall was 97.08%, and the F1 score was 97.09%. For the ResNet152V2 model, accuracy was 96.87%, precision was 96.98%, recall was 97.08%, and the F1 score was 97.09%. In the InceptionV3 classification model, accuracy was 94.19%, precision was 94.44%, recall was 94.07%, and the F1 score was 94.26%. Among the studied classification models, the highest accuracy was obtained in the Xception model, followed by the ResNet152V2, MobileNetV2, and InceptionV3 models, respectively.

The performance of segmentation tests using UNet, SegNet, InceptionUNet, and ENet was evaluated in the segmentation phase for the diagnosis of mandibular furcation defects, and the results are presented in Table 2. The highest Jaccard index value of 96.90% was obtained in ENet segmentation, followed by UNet (94%), SegNet (92.44%), and InceptionUNet (87.64%) segmentation tests (Table 3).

**Table 3:** Performance evaluation metrics and Jaccard indices of the deep learning segmentation models (UNet, InceptionUNet, SegNet, and E-Net) for both healthy and diseased regions in the detection of mandibular furcation defects.

	UNet Segmentation Test Set Results			InceptionUNet Segmentation Test Set Results			SegNet Segmentation Test Set Results			E-Net Segmentation Test Set Results		
Metrics	Mean	Healthy Pixel	Diseased Pixel	Mean	Healthy Pixel	Diseased Pixel	Mean	Healthy Pixel	Diseased Pixel	Mean	Healthy Pixel	Diseased Pixel
**Test Loss**	0.0016	-	-	0.0034	-	-	0.0019	-	-	0.0008	-	-
**Test Accuracy**	99.94%	-	-	99.86%	-	-	99.92%	-	-	99.96%	-	-
**Test Precision**	97.77%	99.96%	95.58%	92.81%	99.93%	95.70%	95.93%	99.96%	91.91%	99.26%	99.98%	98.57%
**Test Sensitivity**	95.89%	99.98%	91.81%	93.10%	99.935	86.29%	95.89%	99.96%	91.83%	97.57%	99.99%	95.30%
**Test F1 Score**	96.82%	99.97%	93.65%	92.96%	99.93%	90.75%	95.91%	99.96%	91.87%	98.41%	99.98%	96.90%
**Jaccard Test Index**	94.00%	99.94%	88.08%	87.64%	99.86%	75.435%	92.44%	99.925	84.96%	96.90%	99.97%	94.00%

The Receiver Operating Characteristic (ROC) curve is a graphical representation based on probabilities, and the Area Under the Curve (AUC) indicates how well the model distinguishes between classes. A higher AUC value reflects better class separation performance. As the system’s effectiveness improves, the AUC increases and approaches one. In this study, the ROC scores reaching up to 99.91% demonstrate that the models possess a high discriminative capability in detecting furcation defects. These results indicate that the proposed models are highly effective in distinguishing defective cases from non-defective ones, highlighting their potential utility in practical diagnostic or quality control applications. In this study, a diseased pixel is defined as any pixel that falls within the anatomical boundaries of the furcation region, where pathological changes are typically observed.

### Visual Evaluation

**Figure 2:**
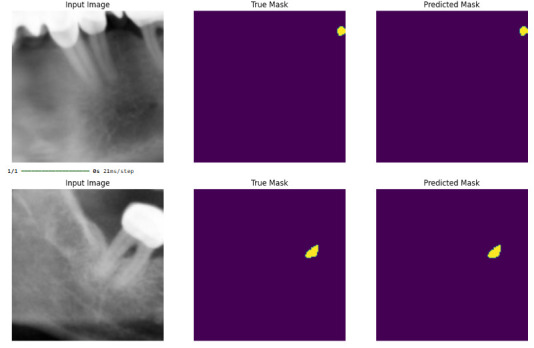
The input image, the real mask, and the mask estimated from selected test examples are presented for ENet.

In Figure 2, the input image, the ground truth mask, and the predicted mask from the test samples are presented together. These steps were repeated in the same manner for each segmentation model. The models demonstrated successful pixel-level predictions, with predicted masks showing strong alignment with the corresponding ground truth. In particular, ENet exhibited robust segmentation performance, as no false positives or false negatives were visually identified in the presented test samples.

## 4. Discussion

In recent years, artificial intelligence (AI) has become increasingly important in medicine and dentistry, with ongoing efforts to minimize diagnostic errors in periodontal assessment.

Convolutional Neural Networks (CNNs) have long been the dominant approach in dental image analysis, demonstrating strong performance across a wide range of tasks. More recently, transformer-based architectures, originally developed for natural language processing, have emerged as promising alternatives for visual tasks, including applications in dentistry.

Several studies have begun to investigate and compare the performance of CNNs and transformers in various dental image analysis tasks [18]. In a study conducted by Schneider, two CNN-based models (U-Net, DeepLabV3+), two hybrid architectures (SwinUNETR, UNETR), and two Transformer-based models (TransDeepLab, SwinUnet) were evaluated across three dental segmentation tasks: teeth, tooth structures, and caries lesions. These tasks were assessed using different imaging modalities, including panoramic and bitewing radiographs. The findings revealed that CNNs significantly outperformed both hybrid and Transformer-based architectures across all tasks and imaging types [19]. In our study, CNN-based models also demonstrated successful performance in the segmentation of furcation involvements.

Although several studies in the literature have investigated the diagnosis of alveolar bone loss [20,21] in periodontal disease using periapical and panoramic radiographs, research specifically focusing on the detection of furcation lesions remains limited. Therefore, the present study aimed to evaluate the effectiveness of artificial intelligence in identifying furcation areas of mandibular molars on panoramic radiographs. The analysis was limited to mandibular molars, as the superimposition of the palatal root in maxillary molars complicates the detection of furcation defects.

In a study evaluating the accuracy, sensitivity, and specificity of panoramic and periapical radiographs in the diagnosis of furcation involvement, no significant difference was observed between the two modalities regarding the detection of furcation defects. The authors suggested that this finding could be attributed to the substantial improvements in the quality of digital panoramic radiographs in recent years [22]. Based on this rationale, panoramic radiographs, which provide the advantages of lower radiation exposure and greater time efficiency, were evaluated in the present study.

In the study conducted by Khan, different semantic segmentation architectures (U-Net, XNet, and SegNet) were employed to detect various dental conditions, including caries, bone loss, and furcation defects, on 206 periapical images [23]. Among all the architectures evaluated on the test dataset, U-Net demonstrated the best performance. Due to the presence of multiple classification categories in their study, the average Jaccard index (mIoU) was reported as 0.402 [23]. In this study, only furcation defects were classified, and when the UNet model was used, the calculated Jaccard index was 94%. Although this study is similar to ours in that it applies segmentation methods for the detection of periodontal bone loss, it does not include a detailed evaluation of the imaging techniques used or the specific characteristics of the bone destruction patterns. While Khan et al. conducted their study using only 206 masked images, our study employed a considerably larger dataset consisting of 2096 masked images. It is believed that the increased dataset size enhanced the model’s generalization ability and positively impacted the Jaccard score.

In a study conducted by Vilcomir et al., deep learning was employed to classify mandibular molar furcation involvement in periapical radiographs. The ResNet-18 model was reported to classify healthy and furcation-involved molars in the test set with an accuracy of 96.47% [24]. In our study, one of the deep learning algorithms used, ResNet152V2, achieved a similar accuracy of 96.87%, comparable to the findings of Vilcomir et al. Furthermore, this accuracy increased to 97.09% when the classification model Xception was used and further rose to 99.96% in segmentation tests.

In a study by Kurt-Bayrakdar al., deep learning algorithms were utilized to detect periodontal bone loss patterns and furcation defects on panoramic radiographs. Their architecture demonstrated the highest diagnostic performance for alveolar bone loss and the lowest for vertical bone loss. For furcation defects, specifically, sensitivity, precision, F1-score, and accuracy, values of 89.2%, 93.3%, 91.2%, and 83.7%, respectively, were reported [25]. While the classification models in the present study yielded comparable results, our ENet segmentation model achieved substantially higher performance metrics, with values of 97.57%, 99.26%, 98.41%, and 99.96%, respectively, representing the highest performance obtained in our study.

Krois et al. employed a convolutional neural network (CNN) to detect periodontal bone loss on panoramic radiographs and compared the system’s performance with the assessments of six dentists [12]. The authors reported accuracy, specificity, and sensitivity values of approximately 81%. Although the bone loss patterns investigated in their study differed from those evaluated in the present study, the accuracy rates obtained from our deep learning models (94–99%) were considerably higher. However, an important strength of the study by Krois et al. was the inclusion of multiple expert clinical evaluations, providing a broader clinical validation framework.

Similarly, Kim et al. utilized the DeNTNet system to evaluate periodontal bone loss on panoramic radiographs and compared the AI-based assessments with clinician performance. While clinicians achieved an average F1-score of 69%, the AI system outperformed them with an F1-score of 75% [26]. In the present study, the F1-scores obtained from different deep learning architectures were markedly higher, ranging from 92% to 98%.

Furthermore, Jiang et al. classified periodontal bone loss into vertical, horizontal, and furcation defect categories using 640 panoramic radiographs and compared AI performance with that of dentists. The authors reported an F1-score of 83% for the furcation defect category, whereas our models achieved F1-scores ranging from 92% to 98% [27]. The superior performance observed in the present study may be attributed to the utilization of more advanced and diverse architectures, including Xception and ENet. Nevertheless, despite these promising results, the interpretability and clinical generalizability of our findings remain limited by the absence of direct comparisons with manual assessments performed by multiple experienced clinicians. Future studies incorporating multi-expert clinical validation are therefore warranted.

Since the furcation defect can be diagnosed radiographically by the presence of a triangular radiolucency in the furcation region and/or when the bone level falls below the furcation region, the radiographic image can sometimes be confusing. Therefore, the disease requires a process that includes clinical examination as well as radiographic imaging. In this study, since furcation involvement in maxillary molars is more difficult to detect on panoramic radiographs, we focused on the detection of furcation involvement in mandibular molars with AI. Including maxillary molar FI detection in future AI studies will provide a more comprehensive understanding of the benefits of AI in diagnosing furcation involvement in both maxillary and mandibular molars. Although panoramic radiographs, which are preferred in this study due to their widespread use in dental radiographic examinations, have various advantages, the quality and magnification of the images may vary depending on the patient’s position [28]. Therefore, to achieve significantly high accuracy in the clinical application of deep learning, it is important to use in-hospital or hybrid datasets from multiple machines and situations [29]. The fact that the panoramic radiography images evaluated in this study were obtained from different devices at the Faculty of Dentistry of Ankara University may be an effective factor in the high accuracy rates obtained in the study. In addition, the segmentation method we used is much more advantageous because it provides more detailed information for evaluating disease severity and the treatment planning process by determining the defect area with its boundaries. In this method, the defect area is processed as a detailed map, thus providing a visually more advanced diagnostic support. The higher accuracy rates obtained in the segmentation test results provide evidence for this situation. A key limitation of this study is the lack of external validation using datasets from different institutions or imaging devices. Future studies should incorporate multi-center datasets to evaluate the generalizability and robustness of the proposed models.

The use of artificial intelligence (AI) systems for interpreting dental radiographic images holds great promise in diagnosis and treatment planning. AI systems can detect subtle details that may be overlooked by dentists due to factors such as fatigue or cognitive overload, and can serve as a decision-support mechanism in cases where diagnosis is challenging due to a lack of experienced clinicians. When the literature is examined, although studies on the detection of furcation involvement are limited, it has been seen that the use of AI systems has yielded successful and promising results. To the best of our knowledge, our study is the first to employ different deep learning algorithms and segmentation methods in the detection of furcation involvement. However, only furcation involvement in mandibular molars was examined in our study. Including larger datasets that encompass maxillary molar teeth is likely to enhance the success rates in detecting furcation involvement.

## 5. Conclusions

In conclusion, despite the limitations of our study, the evaluated data and the obtained findings indicate that artificial intelligence-based decision support systems, especially deep learning algorithms and segmentation tests, can serve as a valuable tool in the detection of furcation involvement in mandibular molars on panoramic radiographs.

## Figures and Tables

**Table 1:** Data splitting details.

Split	Furcation-Positive Crops	Furcation-Negative Crops	Total Classification Crops	Segmentation Image–Mask Pairs
Training, 70%	1467	1467	2934	1467
Validation, 20%	419	419	838	419
Testing, 10%	210	210	420	210
Total	2096	2096	4192	2096

**Table 2:** Performance measures of classification models.

	Xception Classification Test Set Results	ResNet152V2 Classification Test Set Results	MobileNetV2 Classification Test Set Results	InceptionV3 Classification Test Set Results
**Test Loss**	0.07	0.08	0.07	0.15
**Test Accuracy**	97.09%	96.87%	96.42%	94.19%
**Test Precision**	97.10%	96.985	96.59%	94.445
**Test Sensitivity (recall)**	97.08%	96.72%	96.44%	94.07%
**Test F1 Score**	97.09%	96.58%	96.52%	94.26%
**Test ROC**	99.51%	99.70%	99.91%	99.19%

## Data Availability

The panoramic images that support the findings of this study are available from Ankara University Faculty of Dentistry Radiology Department, but restrictions apply to the availability of these data, which were used under license for the current study, and so are not publicly available. The datasets and resulting graphs generated during the current study are available from the corresponding author on reasonable request.

## Abbreviations

The following abbreviations are used in this manuscript:

AI	Artificial intelligence
FD	Furcation defect
ROC	Receiver operating characteristic
AUC	Area under the curve
TP	True Positive
FP	False Positive
TN	True Negative
FN	False Negative
CNNs	Convolutional Neural Networks
